# Neural Correlates of Variations in Human Trust in Human-like Machines during Non-reciprocal Interactions

**DOI:** 10.1038/s41598-019-46098-8

**Published:** 2019-07-10

**Authors:** Eun-Soo Jung, Suh-Yeon Dong, Soo-Young Lee

**Affiliations:** 1 School of Electrical Engineering, Korea Advanced Institute of Science and Technology, Daejeon, Republic of Korea; 2 Department of Information Technology Engineering, Sookmyung Women’s University, Seoul, Republic of Korea; 3 Institute for Artificial Intelligence, Korea Advanced Institute of Science and Technology, Daejeon, Republic of Korea; 4 Brain Science Research Center, Korea Advanced Institute of Science and Technology, Daejeon, Republic of Korea; 5 Present Address: Samsung SDS, Seoul, Republic of Korea

**Keywords:** Cooperation, Human behaviour

## Abstract

As intelligent machines have become widespread in various applications, it has become increasingly important to operate them efficiently. Monitoring human operators’ trust is required for productive interactions between humans and machines. However, neurocognitive understanding of human trust in machines is limited. In this study, we analysed human behaviours and electroencephalograms (EEGs) obtained during non-reciprocal human-machine interactions. Human subjects supervised their partner agents by monitoring and intervening in the agents’ actions in this non-reciprocal interaction, which reflected practical uses of autonomous or smart systems. Furthermore, we diversified the agents with external and internal human-like factors to understand the influence of anthropomorphism of machine agents. Agents’ internal human-likenesses were manifested in the way they conducted a task and affected subjects’ trust levels. From EEG analysis, we could define brain responses correlated with increase and decrease of trust. The effects of trust variations on brain responses were more pronounced with agents who were externally closer to humans and who elicited greater trust from the subjects. This research provides a theoretical basis for modelling human neural activities indicate trust in partner machines and can thereby contribute to the design of machines to promote efficient interactions with humans.

## Introduction

Technological advances have extended the applications of intelligent machines, and humans therefore have more opportunities to cooperate with machine partners within a team. Trust can lead a team to successful cooperation, and teams of humans and machines are no exception. Human operators’ appropriate trust in partner machines is critical for their efficient cooperation^1–4^. An operator’s distrust in machines can lead an operator to frequently intervene and make the machines useless, and over-trust can result in severe mistakes in automation. However, human users’ self-reporting of trust during interactions with machines is inefficient and can be unreliable because the reports may not be sincere or because an individual may be biased against particular criteria. Therefore, understanding neurocognitive responses related to human trust in machines and engendering appropriate trust from humans are important in the development and application of intelligent machines.

Human trust in machine partners has different characteristics from trust in human partners^1,5–7^. Therefore, instead of focusing on factors identified in trust between humans^8^, we focused on human-likeness of machines as a factor for human trust in machines^9,10^. Humans tend to expect automated agents to be perfect and thus are less tolerant of mistakes than they are of mistakes made by humans^5^. Namely, human trust in automation can more easily be broken due to humans’ higher expectations of automation. Research that assigned anthropomorphism to automated agents also demonstrated that agents with enhanced humanness gained more resilient trust^9^ and were blamed less for mistakes^10^. Additionally, there are human-like factors of machines that affect human trust in machines but work differently on individuals according to their characteristics^11^.

Studies using functional magnetic resonance imaging (fMRI) have demonstrated different brain activation in response to untrustworthy human faces compared with trustworthy faces^12,13^ and investigated the neural correlates of building trust during interactions between humans^14^. Previous research in electroencephalography (EEG) also have provided human brain responses according to participants’ trust in sensor systems for driving^15^ or co-operators in trust games, such as investment game^16,17^ and coin toss guessing^18,19^. A study demonstrated that human-like cues (human face and voice) affect neural responses to a machine partner’s technical capability during a theory-of-mind game^20^. A trust sensor model with EEG and galvanic skin response was proposed and demonstrated the feasibility of psychophysiological measurements of human trust in automation^21^.

Most of the pragmatic applications of automated systems are operated in non-reciprocal interactions where a human supervises the systems. For example, current self-driving system operates under supervision of a driver for safety reasons; a human operator can interfere with the system whenever necessary but not the other way around. However, the neurocognitive aspects of human supervisors’ trust during non-reciprocal interactions with automated agents has not been widely explored. The interaction between subjects and agents in our research was a kind of non-reciprocal interaction, where only subjects could make an action according to agents’ action. To the best of our knowledge, there was no previous attempt to investigate neural correlates of human trust in automated agents during non-reciprocal interactions. In this study, we designed and conducted an experiment for non-reciprocal interactions between humans and machine agents. We measured EEG responses and investigated human neural responses related to the development, maintenance, and degradation of situational or learned trust^22^ in machine teammates and the factors that influence that trust. Especially, we focused on the influences of machine human-likenesses on the partner human’s trust. We hypothesized that human-likenesses of automated agents will have a significant impact on behavioural and neural responses of human supervisors related to trust variations and formations.

## Results

**Figure 1:**
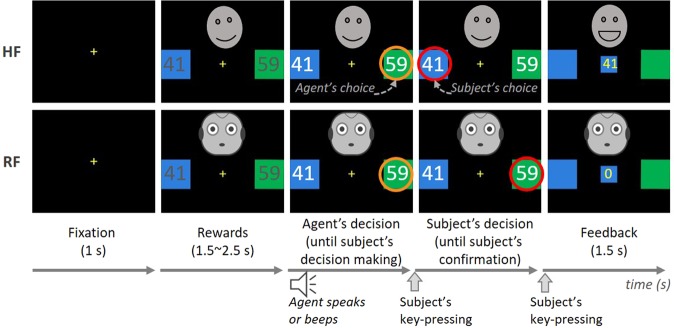
Experimental design. Example trials for HF and RF agents. In each trial, an agent’s face and two rectangles (one blue and one green) are presented after a fixation period. A corresponding reward is marked on each rectangle simultaneously (Rewards stage). A few seconds later, each agent presents its choice with sounds (HF speaks “blue” or “green” in Korean, and RF beeps regardless of blue or green) as the text colour changes to white (Agent’s decision stage). Subjects cannot submit their decisions during the Rewards stage but must wait until the agent presents its decisions, and subjects’ decisions can be different from those of the agent’s. A subject’s decision can be submitted and confirmed by key-pressings, and it is taken as the final decision for each trial. After the submission of a final decision, a trial ends with feedback showing whether the final decision is correct or wrong (Feedback stage). In the example trial with an HF agent, a subject’s final decision is different from that of the agent’s, and the final answer is correct. Consequently, the subject earns the points for the correct rectangle. In the example trial with an RF agent, a subject accepts the agent’s decision, which is wrong, and earns no reward. A face from Japanese Female Facial Expression (JAFFE) database and the face of robot Pepper were modified and used for HF and RF agent, respectively, in our experiment. However, the faces are removed and replaced with emoji in this manuscript due to the image copyright for publication. HF agents make small head movements (tilt slightly to left and right) during the Rewards stage and smile/frown when a final answer is correct/wrong during the Feedback stage. RF agents have no change in their appearance.

An experiment was conducted to record human EEG signals (15 subjects) while performing a decision-making task^23^ with six externally and internally different machine agents (three human-faced (HF) agents with different risk-taking personalities and three robot-faced (RF) agents with different risk-taking personalities). In this task, a subject had to guess a correct colour (either blue or green) to earn points for the correct colour in each trial together with an agent partner. Between the two options represented as colours, one was riskier with higher points than the other. Namely, subjects had to consider ‘how much risk to take for the given rewards’ as the main factor for decision making in the task. Therefore, we adopted risk-taking personalities for agents’ internal human-likeness and designed agents with various risk-taking levels as there are people with different characters. Each agent presented better choices depending on its risk-taking personality, and a subject supervised the agent by monitoring and intervening against the agent’s decisions (details in the Materials and Methods section and Fig. 1). Subjects’ behaviours and EEG signals were analysed and interpreted according to their trust and the agents’ human-likenesses.

### Behavioural responses

Subjects’ evaluations from the questionnaires conducted during the experiment and the number of interventions against each agent’s play are presented (Table 1) and were analysed with respect to the agents’ human-likeness factors. The effects of both external and internal human-like factors on subjects’ questionnaire responses were assessed via a two-way repeated analysis of variance (ANOVA) with two within-subject factors: agents’ external (HF and RF) and internal human-likeness (the level of risk-taking personality of agents: high, medium, and low). The agent’s external human-likeness exhibited significant effects on evaluations of human-likeness (F(1, 14) = 35.79, p < 0.001) and familiarity (F(1, 14) = 11.90, p = 0.0098) but not on risk-taking personality (F(1, 13) = 1.64, p = 0.37, responses for 14 subjects were tested due to the missing response of one subject), ability (F(1, 14) = 0.41, p = 0.67), or trust (F(1, 14) = 0.01, p = 0.92). On the other hand, internal human-likeness influenced only subjects’ judgements of the agents’ risk-taking levels (F(2, 26) = 25.87, p < 0.001, responses for 14 subjects were tested due to the missing response of one subject) and not their judgements of other characteristics (human-likeness: F(2, 28) = 0.47, familiarity: F(2, 28) = 0.34, ability: F(2, 28) = 0.63, and trust F(2, 28) = 0.88, p > 0.1 for all four cases). All the p-values were corrected by false discovery rate. There was no significant effect of the interaction between internal and external human-likenesses on any of the questionnaire item (p > 0.1 for every case). Namely, subjects judged an agent’s human-likeness and familiarity not with the agent’s risk-taking personality but with the agent’s appearance or voice, whereas they assessed an agent’s risk-taking personalities with the agent’s play, regardless of their appearances or voices.

**Table 1:** Average (standard deviations in parentheses) scores from the questionnaire and the number of interventions for each agent.

	Human-faced & high-risk taking agent	Human-faced & medium-risk taking agent	Human-faced & low-risk taking agent	Robot-faced & high-risk taking agent	Robot-faced & medium-risk taking agent	Robot-faced & low-risk taking agent
Human likeness	3.20	3.00	3.07	1.67	1.53	1.67
	(1.21)	(1.07)	(1.10)	(0.82)	(0.74)	(0.90)
Familiarity	3.33	3.27	2.93	2.07	2.33	2.33
	(0.82)	(0.88)	(1.03)	(0.88)	(1.18)	(1.11)
Risk-taking	4.07	3.40	2.60	3.87	2.86	2.67
	(0.70)	(0.51)	(0.83)	(1.06)	(0.95)	(0.82)
Ability	2.93	3.27	3.27	2.93	3.07	3.00
	(0.59)	(0.70)	(0.96)	(1.03)	(1.03)	(1.20)
Trust	2.73	3.00	3.33	2.93	3.13	3.07
	(0.96)	(1.07)	(0.90)	(1.10)	(1.19)	(1.10)
# of interventions	6.33	4.73	4.87	7.33	5.07	5.20
	(5.50)	(3.75)	(3.31)	(6.49)	(4.04)	(3.41)

On average, a subject intervened on an agent 5.58 times (s.d. = 4.54 with n = 45 sessions) per session, and the gap between the maximum and minimum numbers of interventions of each subject was 6.07 (s.d. = 2.91, n = 15 subjects) for HF and 5.47 (s.d. = 3.77, n = 15 subjects) for RF agents. According to trust scores from the questionnaire and the numbers of interventions, agents experiencing fewer interventions tended to gain higher scores on trust. We observed a significant negative correlation between trust scores from the questionnaire and the numbers of interventions enacted on each agent (Spearman’s r = −0.4, p < 0.001, the numbers of interventions for each subject were normalized to zero mean and unit variance because their range was different for each subject). Therefore, we considered the number of interventions on an agent to be an indicator of the implicit trust level of a subject in the agent.

There was no significant effect of agents’ external (F(1, 14) = 1.217, p = 0.289) or internal human-likenesses (F(1.148, 28) = 1.883, p = 0.190 with Greenhouse-Geisser correction^24^) on the number of interventions for overall subjects. However, there was a significant correlation between the number of interventions on HF and RF agents according to their risk-taking personalities (Spearman’s r = −0.69, p < 0.001, Fig. S1). This result can be interpreted that each subject formed trust in agents according to agents’ plays, which were related to risk-taking levels. Even though the level of trust or human-likeness related to risk-taking level cannot be objectively determined for overall subjects, each subject can perceive certain risk-taking agent more trustworthy than others.

We also analysed subjects’ reaction times after agent decision onsets; however, they exhibited no significant correlation with the explicit trust scores (Spearman’s r = −0.14, p = 0.17). The fastest subject reacted within 0.71 s on average (s.d. = 0.28, n = 192 trials of all six sessions), and the slowest reacted within 1.84 s on average (s.d. = 1.18, n = 192 trials); the overall average of the 15 subjects was 1.16 s (s.d. = 0.38, n = 15 subjects).

### EEG analyses for trust increase and decrease

Previous studies have defined the basis of human trust. According to their conclusions, the most dominant bases of trust are ability, persistence, and intention^4,8,20,25–27^. In our research, an agent’s persistence and intention to help human partners were guaranteed and it was informed to subjects before each session. However, an agent’s risk-taking personality were not informed in advance, thus subjects could only notice each agent’s risk-taking personality from its plays and judge its ability from previous results. As subjects learnt an agent’s personality or strategy, the results of the agent’s decision of each trial affected on the next trial. Therefore, trust level can be changed trial by trial and culminated in the final trust in each agent, not formulated at a statistic value from the beginning. Consequently, we hypothesized that subjects adjusted their trust on each agent considering its previous performance, and investigated the brain activities which can reflect the changes of trust in trial-level. We simplified these changes in trust into two phases: trust increase after agents’ correct decisions (ACs) and decrease after agents’ wrong decisions (AWs). Subjects’ brain activities after agent decision onset were analysed with respect to the change in their trust in the partner agents. This was because we expected that this period was involved with immediate judgements of trustworthiness of partners’ actions before subjects’ final decisions were made.

**Figure 2:**
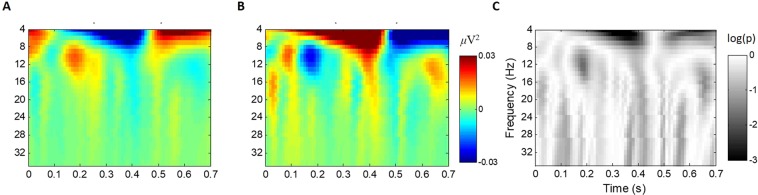
Average EEG power variations in the Cz channel due to trust changes. (**A**) Grand average EEG variations after ACs and (**B**) AWs. (**C**) Log-scaled p-values from two-tailed paired t-tests between AC and AW cases. Average power variations for AC and AW trials of each session are used as samples for paired t-tests (i.e., sample no. = 89 for each case, where a faulty session was discarded from the total of 90 sessions). Data until 0.7 s after the onset are presented because data after 0.7 s are likely to be influenced by subjects’ key-pressings.

To focus on the differences in EEG signals between two consecutive trials with respect to AC and AW cases, a wavelet-transformed EEG signal of the *k*^th^ (*k* < 32) trial was subtracted from that of the (*k* + 1)^th^ trial and grouped according to the performance (correct/wrong) of the agent in the *k*^th^ trial. Studies have uncovered that judgement of trustworthiness is related to the amygdala^12,13^, paracingulate cortex, and ventral tegmental area^14^. Moreover, there are EEG studies which have investigated the neural correlates of trust by observing midline central electrodes^15,16^. Therefore, brain signals from the central region (Cz channel) were first examined (Fig. 2). The results demonstrated that the theta band (4~8 Hz) power at approximately 0.4 s decreased after ACs and increased after AWs. From statistical tests, we can select a continuous time-frequency (TF) region representing EEG responses related to trust decreases and increases (Fig. S2).

**Figure 3:**
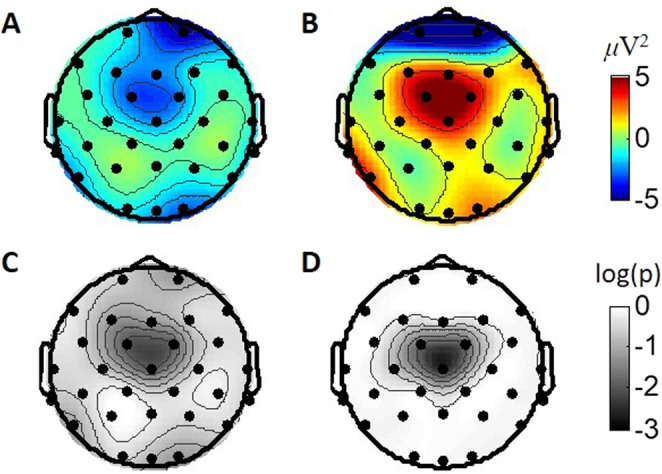
Topographies of average EEG power variations due to trust changes in the selected TF region. (**A**) Grand average of the EEG power changes after AC and (**B**) AW cases, and (**C**) log-scaled p-values (corrected) from two-tailed t-tests for AC and (**D**) AW cases. Average power variations for AC and AW trials of each session are used as samples and compared to zero in t-tests (i.e., sample no. = 89 sessions for each case). Values between electrodes are interpolated.

Observation of EEG power variations between consecutive trials over the selected TF region was extended to all scalp channels. After trials with ACs, subjects tended to produce relatively smaller powers in the theta band in the fronto-central area in response to their agents’ new actions than those they had produced in the previous responses (Fig. 3A), and they produced relatively larger powers after AWs (Fig. 3B). The variations in EEG powers due to AC and AW were proven to be statistically significant (Fig. 3C,D) by rejecting the null hypothesis that the variations were from a distribution with zero mean at Cz (t(88) = −3.54, p = 0.011 for ACs and t(88) = 4.12, p = 0.003 for AWs), FC1 (t(88) = −3.51, p = 0.011 for ACs and t(88) = 3.21, p = 0.019 for AWs), and FC2 (t(88) = −3.31, p = 0.012 for ACs and t(88) = 3.37, p = 0.017 for AWs, all p-values were corrected for multiple hypothesis testing^28^) channels. Our results are also consistent with previous research that demonstrated differences between brain activations due to implicit agreement and disagreement in the fronto-central region^29^. In summary, we defined a TF region of brain responses related to trust variations that were more pronounced in the fronto-central brain area.

To verify that these brain responses are not related to other factors such as disappointment due to failures of achieving points, we additionally analysed subjects’ brain responses during a control experiment where subjects conducted the same task without any agent (details are in the Materials and Methods section and Fig. S3). EEG power variations of the selected TF region were observed with the same method as for the sessions with agents, but were grouped according to the differences due to subject’s correct (SC) and wrong (SW) decisions (Fig. S4). The brain activities that we defined to be correlate to trust variations were not correlated to the results of subject themselves’ decisions or disappointment from the failure.

### EEG analyses for agents’ human-likenesses

**Figure 4:**
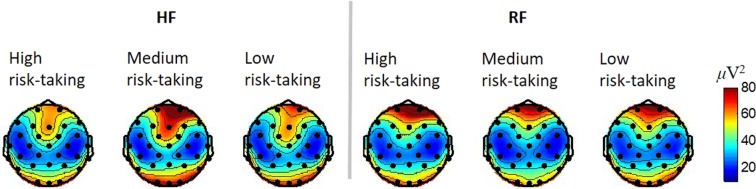
Topographies of average EEG powers for each session. Powers of the selected TF region were observed according to the types of partner agent. Values between electrodes are interpolated.

As in the analyses of subjects’ behaviours, the EEG feature related to trust was also analysed with respect to agents’ external and internal human-likenesses. The powers of selected TF region defined in the previous section were extracted from all trials of each session and averaged (Fig. 4). The brain activation for each session was similar to each other. The powers in the fronto-central channels (FC1, FC2, and Cz) were relatively larger for sessions with HF agents, regardless of risk-taking levels (mean and standard deviations of powers for HF agents: 49.59 ± 23.72 μV^2^, for RF agents: 37.14 ± 9.79 μV^2^). A two-way ANOVA was conducted that examined the effect of external human-likeness (agent types; HF and RF) and internal human-likeness (risk-taking levels; high, medium, and low) on the powers of selected TF region. There was a statistically significant effect of agent types on the powers of selected TF region, F (1, 83) = 10.15, p = 0.002. However, there was no significant effect of risk-taking levels (F(2, 83) = 0.11, p = 0.90) or the interaction between two factors (F(2, 83) = 0.57, p = 0.57). We regarded that the observed brain activations were affected by the audio-visual stimuli for HF and RF agents, and this indicates the necessity of separate observations for EEG changes related to trust variations in HF and RF agents.

### EEG analyses for agents’ external human-likenesses and subjects’ trust levels

In this section, we conducted observation of brain responses related to trust variations separately according to agents’ external human-likenesses and session-level trust defined from our behavioural results. From the behavioural results, the number of interventions against agents’ plays was correlated with subjects’ final trust in agents. Namely, higher trust can be built during sessions where subjects rejected agents’ decisions less often, and subjects’ rejections were affected by agents’ risk-taking personalities. Among three sessions for each subject and agent face type (HF/RF), the one (or ones) with the maximum number of interventions was defined as the low trust session and the one (or ones) with the minimum number of interventions was defined as the high trust session. Not exactly two out of the three sessions were used because there could be multiple sessions with the most/least interventions. One or more sessions were used as high or low trust sessions. Three subjects had two sessions with the maximum/minimum number of interventions among their HF sessions and seven subjects had two sessions with the maximum/minimum number of interventions among their RF sessions.

**Figure 5:**
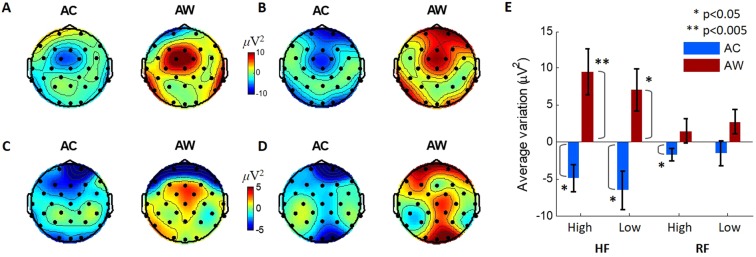
Topographies of average EEG power variations due to trust increase and decrease in the selected TF region presented separately for agent types (HF/RF) and subjects’ trust levels (high/low). Average EEG power variations after AC and AW cases for (**A**) sessions with highly trusted HF agents, (**B**) with low-trusted HF agents, (**C**) with highly trusted RF agents, and (**D**) with low-trusted RF agents. (**E**) Average of EEG power variations in the fronto-central (FC1, FC2, and Cz) brain area (n = 15 subjects for each bar). Average power variation for each case was compared to zero in a t-test. The error bars represent standard errors.

EEG power differences between consecutive trials for AC and AW cases were observed with the same TF range as in the previous section, but they were analysed separately according to the final trust level of each session (Fig. 5A–D). The similar EEG features associated with trust decreases and increases were detected for different sessions with respect to agents’ external human-likenesses and trust levels. In the brain responses to agents’ decisions after ACs, negative changes in theta band power were observed in the fronto-central area, and positive changes were observed after AWs. Thus, we narrowed our observations down to three channels in the fronto-central region (Cz, FC1, and FC2). The power variations over the selected TF region for these three channels were averaged for each subject and tested for significance in the AC and AW cases (Fig. 5E). The statistical significance was confirmed by rejecting the null hypothesis that the variations were from a distribution with zero mean. The trends in the subjects’ EEG power in the selected TF and spatial region were consistent across the external human-likenesses and subject trust levels. However, the trends were more statistically significant for sessions with HF (${\rm{t}}(14)=-\,2.64,\,{\rm{p}}=0.010$ for ACs and ${\rm{t}}(14)=3.05,\,{\rm{p}}=0.004$ for AWs of highly trusted HF agents, and ${\rm{t}}(14)=-\,2.46,\,{\rm{p}}=0.014$ for ACs and ${\rm{t}}(14)=2.44,\,{\rm{p}}=0.014$ for AWs of low-trusted HF agents, all are one-tailed t-tests). Responses related to trust variations were distinctive regardless of the final trust levels in HF agents. However, subjects’ brain responses tended to be less influenced by their RF partners’ performances, especially when they did not build up high trust in the partners (${\rm{t}}(14)=-\,2.00,\,{\rm{p}}=0.033$ for ACs and ${\rm{t}}(14)=0.89,\,{\rm{p}}=0.194$ for AWs of highly trusted RF agents, and ${\rm{t}}(14)=-\,0.90,\,{\rm{p}}=0.192$ for ACs and ${\rm{t}}(14)=1.63,\,{\rm{p}}=0.063$ for AWs of low-trusted RF agents, all are one-tailed t-tests). Moreover, a three-way ANOVA was conducted to examine the effect of external human-likenesses (HF and RF), final trust levels (high and low), and agents’ performances (AC and AW) on neural responses, i.e., the selected TF powers. We could find the statistically significant effect of agents’ performances on neural responses (F(1,112) = 33.34, p < 0.001), but other factors were not statistically significant (external human-likeness: F(1,112) = 0.47, p = 0.50; final trust level: F(1,112) = 0.19, p = 0.66). Also, there was a statistically significant interaction between the effect of agents’ performances and external human-likenesses (F(1, 112) = 11.19, p = 0.001). Post-hoc analysis indicated that the EEG power changes were significantly different between sessions with HFs and RFs for AW cases (p = 0.005), and less significant for ACs (p = 0.062). Additionally, the difference between AC and AW was significantly larger with the HF agents (p < 0.001) than with the RF agents (p = 0.089, all p-values for post-hoc were corrected with Bonferroni correction). Thus, we can interpret these results that subjects’ neural responses were affected by agents’ performances and these EEG changes further enhanced by agent’s external human-likenesses. Together with behavioural results, these results indicate that subjects were less sensitive to the participation of less trusted agents if they were externally less like human.

## Discussion

To understand brain responses related to human trust in machines, we observed subjects’ behaviours and EEG activities while supervising partner agents. We focused on human brain activities related to trust and its formation through multiple trials of interactions with machine agents. As previous research that investigated human trust in anthropomorphic agents or machines^5,9,10^, we hypothesized that machine agents’ human-likenesses can cause significant impacts on human supervisors’ trust. Therefore, two aspects of human-like factors were assigned to each agent: one factor was external human-likeness involving appearance, voice, and movements, and the other was internal human-likeness involving risk-taking trait, which is the main factor in conducting task in our experiment.

Subjects’ explicit judgements of agents’ human-likenesses and familiarities were only affected by their external human-like cues. Each subject’s implicit trust in agents could be defined using the number of interventions in the agents’ decisions, which were influenced by agents’ risk-taking personalities. During the experiment, however, subjects’ trust levels could be changed as they learned the task and the agents’ characteristics. Without sufficient information of an agent’s risk-taking personality or technical competency, subjects could not yet establish their trust in the agent at the beginning of each session. According to subjects’ behavioural results, subjects’ final trust formed after each session was influenced by agents’ risk-taking personalities; each subject had sessions with relatively higher and lower trust due to agents’ different risk-taking personalities.

As technical competence is one of the major factor in trust^4,8,25–27^, subjects’ trust could increase after AC and decrease after AW trials. To understand the formation of trust in a machine agent during each session, we observed the differences between the brain signals elicited during consecutive trials with respect to agent performances. There were significant changes in the theta band power of signals from the fronto-central region at 0.4 s after an agent’s decision onset, in accordance with trust changes. These observations are consistent with previous studies exploring human brain activities related to the evaluation of trustworthiness in faces^12,13^ or implicit intentions of agreement/disagreement in sentences^29^.

We continued the EEG analyses with divided sessions according to subjects’ trust (low and high), which was affected by agents’ internal human-likenesses, and agents’ external human-likenesses (HF and RF). The effects of trust changes were relatively less prominent for RF agents, especially for the sessions where relatively low trust was built. This implied that subjects were less dependent and participated more actively when they did not trust the decisions of their partner agents and therefore paid less attention to the actions and performances of the agents. The formation of trust in externally less human-like agents with risk-taking characteristics that contradicted their human partners was less successful. Subjects’ trust in machine agents can be built up during cooperation, and results of this research provide the neurophysiological mechanisms behind human trust formation. There was no direct relation between each subject’s intervention after a trial with AC or AW, such as less interventions in trials right after an AC or more after an AW, and this is not against our hypothesis or analyses in the result section. An agent’s performances affected on a subject’s trust variation in trial-level, but were not directly concluded into the subject’s decision to trust or distrust the agent in each trial. In this study, we focused on human supervisors’ trust which was built during interactions with their partner agents. Based on the findings from this research, we can extend the scope of human trust mechanism from the beginning to the end of interactions with machines. Future research will focus on trust dynamics modelling with further investigating of the quantitative measure of trust varying in time.

In conclusion, we analysed human behaviours and EEG signals to identify the neurocognitive responses related to trust in non-reciprocal interactions with machines. The interaction involving with decision made by the automated system can be practical application of our research. More common automated decision support systems such as artificial intelligent secretary may be available, as well as professional applications that require risky decisions, such as autopilot and anti-warfare systems. Furthermore, we designed human-like agents with external and internal human-like aspects to explore factors that influence trust. Features in EEG signals indicating changes in human trust in partner machines were demonstrated. This research provides a theoretical basis for the feasibility of monitoring and modelling human trust in machine partners with brain responses, and thus can contribute significantly to the design of machine partners for various applications and to efficient interactions between humans and machines.

## Materials and Methods

### Subjects

We recruited 15 subjects (6 females) who were right-handed, were native Korean speakers, had normal or corrected-to-normal vision, and had no history of psychiatric or neurological disorders. Experimental designs and procedures were all approved by the Institutional Review Board of Korea Advanced Institute of Science and Technology (KAIST) and conducted in accordance with the relevant guidelines and regulations. Written informed consent was obtained from every subject. The ages of the subjects ranged from 21 to 34 years (mean age of 25.1 years and a variance of 3.4 years). Payment for each subject was approximately 20,000 Korean Won for an hour of participation.

### Experimental procedure

Subjects performed a one-armed bandit task (modified from the study of Behrens *et al*.^23^) together with various human-like machine partners to earn additional rewards. In our experiment, machine agents repeatedly chose either blue or green rectangles that had associated rewards, and human subjects supervised the agents by confirming or intervening in their choices (Fig. 1). One of the two rectangles was correct in each trial, and a subject was rewarded with the points on the correct rectangle only when his/her guess was correct. The green rectangle always contained larger rewards but had smaller probability of being correct than the blue rectangle (the probability for blue was 75% and that for green was 25%). The same pair of rewards was not presented more than once in each session, but the sum of every reward pair was 100 for each trial. Subjects were informed that the probability that the blue rectangle was correct was always higher than that of the green one but were not told the exact probability. In each trial, a subject submitted a final choice between the two options by accepting or rejecting a partner agent’s decision, and then the outcome of the choice was revealed as feedback. Therefore, subjects could update their strategies using the results of previous trials, for example, changing how much risk to take, or how much to trust their partner agents. Each session consisted of 32 trials, which were 32 decision-making trials. Each subject participated in six overall sessions with a different agent in each session. The order of the 32 reward pairs in each session was pseudo-random (same random order for all subjects). Furthermore, the order of the six sessions was random, except that there were not three sessions with HF or RF in a row. The order of sessions was randomly permuted for each subject with MATLAB (The MathWorks, Inc., Natick, MA, USA). Subjects performed each session within five minutes. A questionnaire about each agent was given to subjects after every session, and each subject scored (from 1, lowest, to 5, highest) each agent’s human-likeness, familiarity, risk-taking level, and trust (Table 1). After the experiment, a bonus was paid to each subject in proportion to the points obtained during the whole experiment.

Unlike subjects, machine agents had information about the probability that each rectangle was correct and evaluated the two options in consideration of the information and given rewards. As humans would make decisions that consider the tradeoffs between a given option’s payoffs and risks, we designed agents with various risk-taking levels by controlling a risk-taking parameter modelled in the study of Behrens *et al*.^23^. This risk-taking personality was a factor of human-likeness that we controlled in our experiment. All agents have this personality but differently; we designed different agents with risk-taking levels as there are people with different characters. Different agents calculated the value differently with respect to their risk-taking personalities. Each agent evaluated an option using the following equations:

$${g}_{{\rm{blue}}}=F({r}_{{\rm{blue}}},\,\gamma )\cdot {f}_{{\rm{blue}}}$$

$$F({r}_{{\rm{blue}}},\gamma )=\,{\rm{\max }}[{\rm{\min }}[\gamma ({r}_{{\rm{blue}}}-0.5)+0.5,1],0]$$

where ${f}_{{\rm{blue}}}$ is the reward size of the blue rectangle (different for each trial), ${r}_{{\rm{blue}}}$ is the probability that blue was correct, and $\gamma$ is a risk-taking parameter. Each agent calculated the value ${g}_{{\rm{blue}}}$ and ${g}_{{\rm{green}}}$ and chose the one with the bigger value. We set three risk-taking levels with $\,\gamma =0.7$, 1, and 1.5 for high, medium, and low risk-taking, respectively; there were two agents with each risk-taking level. A pair of options was given in each trial, and an agent selected the better choice with its own criteria as described above. As risk-taking personality is an internal human-likeness factor, subjects could notice agents’ risk-taking level only by performing the task with them. Experimental settings were controlled so that each agent could earn a similar level of reward (agents themselves could achieve 75.1~77.2% of the total rewards without human supervisors). Subjects were informed of the ability of the agents and their intentions to help subjects, and thus subjects could build trust as they interact with the agents.

In addition to risk-taking personality traits, there was another factor that made the machine agents distinctive. This factor could be externally recognized with audio-visual representations of the agents. The six agents in our experiment can be classified into two types according to this external human-likeness: human-faced (HF) and robot-faced (RF) agents. HF agents had a human female face, female voice, movements, and facial expressions. A happy Asian female face^30^ was used with modifications because previous research concluded that humans tend to trust more in happy faces than angry or sad faces^12^. We recorded a female voice speaking “blue” and “green” words in the Korean language and played a recording at each agent’s decision stage in sessions with the HF agent. RF agents had robot Pepper’s face (SoftBank Robotics, Japan) and could make a “beep” sound but no movement. We also adopted expressing emotions with facial expressions (smiling and frowning) as a factor of external human-likeness. Therefore, we designed HF agents to smile or frown according to a result of each trial, but not the robot-faced agents.

In summary, there were six machine agents; each conducted a one-armed bandit task with subjects in each session. Every agent was unique, with different external and internal human-likenesses. Each agent’s external human likeness was audio-visually revealed, whereas the internal human likeness (or risk-taking personality) could be only perceived from observing its plays in each session. An agent’s decision can be accepted as the final decision or rejected with an intervention of a subject in each trial.

Additionally, we had conducted an additional control session during our experiment for each subject (Fig. S3). In this session, subjects had to perform the same one-armed bandit task without any agent. Neither did an agent nor its decision was appeared, thus subjects had to make decisions by themselves. The experimental settings, such as the number of trials or the probability of each colour to be correct were same as the other sessions with agents.

### EEG recording and preprocessing

The EEG signals were recorded using BrainCap (Brain Products GmbH), which has 32 integrated electrodes (Ag/AgCl and passive) located at standard positions given by the International 10–20 system^31^ (Fig. S5), and BrainAmp (Brain Products GmbH) in an electromagnetically shielded room. Among the thirty-two channels, thirty were for scalp potentials, one was for Electrooculogram (EOG), and another was for Electrocardiogram (ECG). Individual sensors were adjusted under 20 kΩ impedance during the whole period of each experiment. The sampling rate was 500 Hz, and a notch filter at 60 Hz was used during the measurement. The signals were recorded using BrainVision Recorder 1.10 and exported using BrainVision Analyser 1.05 (Brain Vision LLC).

A MATLAB toolbox, EEGLAB^32^, was used for preprocessing. First, the reference was transformed from the FCz channel to an average-reference to reduce the effects of the original reference-site activity on other EEG channel^32–34^. Second, artefacts due to eye movements and heartbeat were reduced using independent component decomposition (ICA)^32,35^ based on the extended Infomax algorithm^36^. Components to reject were selected manually. Third, the signals were filtered using a high-pass filter with a cutoff frequency of 0.95 Hz and a low-pass filter with a cutoff frequency of 35 Hz. The high-pass filter removed linear trends with very slow voltage changes^32,33^, whereas the low-pass filter reduced artefacts from electromyography (EMG)^33,37^.

EEG data from each trial were epoched from −200 ms to 700 ms relative to each agent’s decision onset, where the mean EEG amplitude during the 200 ms before the onset was used as the baseline for each trial and channel. Time-frequency representations of spectral power between 4 and 35 Hz were obtained with complex Morlet wavelet transformation^38^ with a central frequency at 1 Hz intervals. No normalization was applied to our analyses because our observation was focused on power differences between trials and normalization could have weakened these differences.

### Statistical analysis

The two-way repeated measure ANOVA tests in the Behavioural Response section were performed using IBM SPSS Statistics 21 (IBM Corp. Armonk, NY). The sphericity of each distribution for the ANOVA test was verified by Mauchly’s sphericity test^39^ with a threshold of p < 0.05. For a case whose sphericity was not supported by Mauchly’s test, the degree of freedom was adjusted by Greenhouse-Geisser’s epsilon^24^. For comparisons in each within-subject effect, p-values were corrected using the method of false discovery rate (FDR) for multiple comparisons. To select TF regions for EEG differences between ACs and AWs, clusters consisting of adjacent bins that all exceed a threshold^40,41^ of p < 0.05 and covering at least one frequency band were considered significant. In EEG Analyses for Trust Increase and Decrease section, p-values were also FDR-corrected for multiple hypothesis testing introduced in the study of Storey^28^. The ANOVA tests for EEG analyses were also performed using IBM SPSS Statistics 21. For significant interaction, post-hoc pairwise comparisons with Bonferroni corrections were carried out.

## Supplementary information

Supplementary Information for Neural Correlates of Variations in Human Trust in Human-like Machines during Non-reciprocal Interactions

## Data Availability

The data from our experiment are available from the corresponding author upon request. The data are not publicly available according to the policy protecting subjects’ personal data.
