# Supplementary material for: Neural Correlates of Variations in Human Trust in Human-like Machines during Non-reciprocal Interactions
Source: Sci Rep. 2019 Jul 10;9:9975. doi: 10.1038/s41598-019-46098-8 (PMC6620272; doi:10.1038/s41598-019-46098-8)
Supplement: Supplementary file 1 — Supplementary Information for Neural Correlates of Variations in Human Trust in Human-like Machines during Non-reciprocal Interactions [file 41598_2019_46098_MOESM1_ESM.docx]

Supplementary Information for

Neural Correlates of Variations in Human Trust in Human-like Machines
during Non-reciprocal Interactions

Eun-Soo Jung^+^, Suh-Yeon Dong^+^, Soo-Young Lee^*^

^+^ These authors contributed equally to this work.

* Correspondence to: Soo-Young Lee (sy-lee@kaist.ac.kr)

**This file includes:**

Figs. S1 to S5

**Figure S1**. Correlation between the number of interventions for HF and RF agents of the same risk-taking personalities. Each marker (n=45, three for each subject) presents the number of interventions of a subject for HF and RF agents with the same risk-taking personality (high/medium/low); a marker’s x-coordinate represents the number of interventions of a subject for an RF agent and y-coordinate represents the number of interventions of the subject for the HF agent with the same risk-taking level as the RF agent (e.g., the x- and y-coordinates of a sample indicate a subject’s interventions for *high*-risk taking RF and that for *high*-risk taking HF agent, respectively). Noise was added to each point to avoid overlaps of multiple markers. Dotted line corresponds to the best-fit regression model.


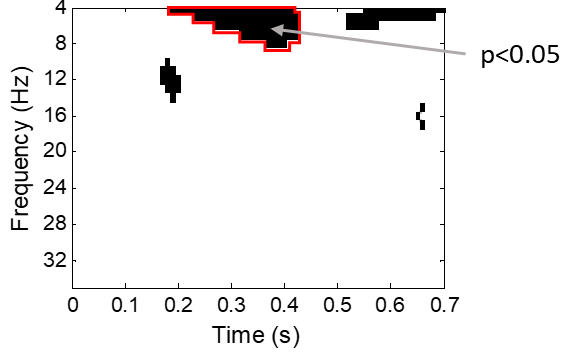


**Figure S2.** Selected TF regions representing significant EEG power differences due to trust changes. From the result of Fig. 2C, only TF bins with p<0.05 are filled. For further analyses, a region of a cluster consists of adjacent bins that all exceed a threshold of p<0.05 in the theta band was selected (n=74 bins in the red boundary), and this TF region includes bins with a false detection rate with corrected p-values less than 0.05.


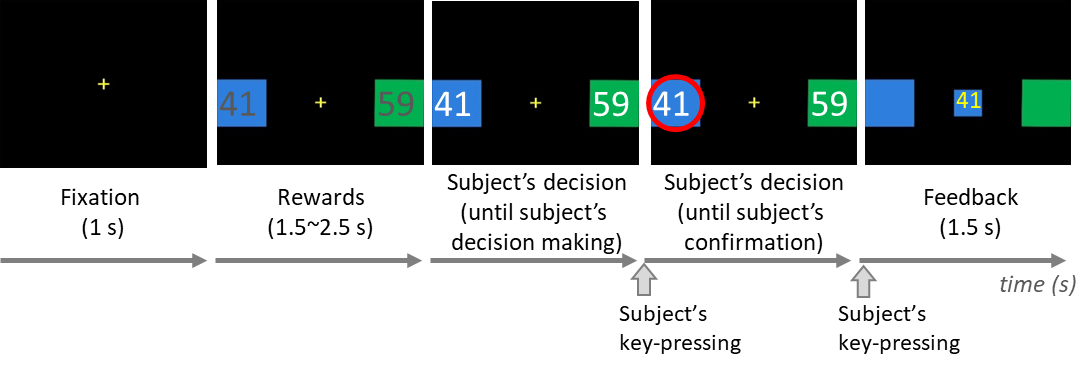


**Figure S3**. Experimental design for control session without agent. In this session, subjects had to perform the same one-armed bandit task without any agent. In this example, the subject chose the blue rectangle, which is correct.


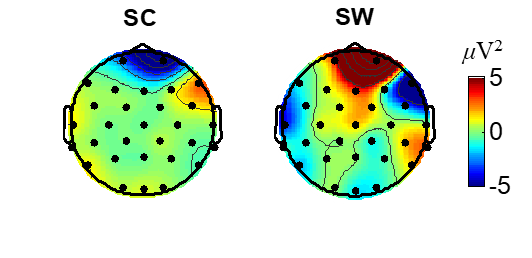


**Figure S4.** Topographies of average EEG power variations due to subjects’ decisions. EEG power variations of the selected TF region from Fig. S2 were observed with the same method as for the sessions with agents, but were grouped according to the differences due to subject’s correct (SC) and wrong (SW) decisions. There were no statistically significant changes with respect to SC and SW in any of channels. The average of EEG power variations in the fronto-central (FC1, FC2, and Cz) brain area could not reject the null hypothesis that the variations were from a distribution with zero mean for both groups (SC: t(14) = -0.18, p=0.4 and SW: t(14)=0.83, p=0.2). Namely, the brain activities that we defined to be correlate to trust variations were not correlated to the results of subject themselves’ decisions or disappointment from the failure. Values between electrodes are interpolated.

**Figure S5**. Locations of 30 scalp electrodes used in this research. Additionally, the EOG electrode was placed below a subject’s left eye, and the ECG electrode was placed on the left collarbone. The reference channel was on FCz, which was located between the Fz and Cz electrodes.
